# *Leishmania* (*Sauroleishmania*) *tarentolae* isolation and sympatric occurrence with *Leishmania* (*Leishmania*) *infantum* in geckoes, dogs and sand flies

**DOI:** 10.1371/journal.pntd.0010650

**Published:** 2022-08-09

**Authors:** Jairo Alfonso Mendoza-Roldan, Andrea Zatelli, Maria Stefania Latrofa, Roberta Iatta, Marcos Antonio Bezerra-Santos, Giada Annoscia, Floriana Gernone, Jan Votýpka, David Modrý, Lucie Tichá, Petr Volf, Domenico Otranto

**Affiliations:** 1 Department of Veterinary Medicine, University of Bari, Valenzano, Italy; 2 Interdisciplinary Department of Medicine, University of Bari, Valenzano, Italy; 3 Department of Parasitology, Faculty of Science, Charles University, Prague, Czech Republic; 4 Biology Centre, Institute of Parasitology, Czech Academy of Sciences, České Budějovice, Czech Republic; 5 Department of Botany and Zoology, Faculty of Science, Masaryk University, Brno, Czech Republic; 6 Department of Veterinary Sciences, Faculty of Agrobiology, Food and Natural Resources, Czech University of Life Sciences, Prague, Czech Republic; 7 Department of Pathobiology, Faculty of Veterinary Science, Bu-Ali Sina University, Hamedan, Iran; University of Glasgow, UNITED KINGDOM

## Abstract

The trypanosomatid protist *Leishmania tarentolae* is a saurian-associated parasite vectored by the *Sergentomyia minuta* sand fly. This study aimed to confirm the circulation of *L*. *infantum* and *L*. *tarentolae* in sand flies, reptiles and dogs and to isolate new strains of these protists. Reptilian and sheltered dog blood samples were collected, and sand flies were captured. Samples were tested for *Leishmania* spp. using duplex real-time PCR (dqPCR) and real-time PCR (qPCR); the origin of blood meal was identified in engorged sand flies by conventional PCR. The reptilian blood and intestinal content of sand fly females were cultured. Dog sera were tested by IFAT using both *Leishmania* species. Four *Tarentola mauritanica* geckoes were molecularly positive for *L*. *infantum* or *L*. *tarentolae*, with no co-infections; moreover, amastigote-like forms of *L*. *infantum* were observed in the bone marrow. 24/294 sand flies scored positive for *Leishmania* spp. by dqPCR, 21 *S*. *minuta* and two *Phlebotomus perniciosus* were positive for *L*. *tarentolae*, while only a single *Ph*. *perniciosus* was positive for *L*. *infantum*. Blood meal analysis confirmed reptile and dog in *S*. *minuta*, dog and human in *Ph*. *perniciosus* and dog in *Phlebotomus neglectus*. Two axenic strains of *L*. *tarentolae* were obtained. Twelve of 19 dogs scored positive for *L*. *infantum* and *L*. *tarentolae* by IFAT and three of them also for *L*. *infantum* by dqPCR, and six by qPCR. These data confirm the sympatric circulation of *L*. *infantum* and *L*. *tarentolae* in geckoes, sand flies, and dogs, and suggest that geckoes may be infected with *L*. *infantum*.

## Author summary

*Leishmania tarentolae* is a saurian-associated parasite recently reported in geographical areas where *Leishmania infantum* is endemic. To confirm the circulation of both protists in sand flies, reptiles and dogs and to isolate new *Leishmania* spp. strains, reptilian and sheltered dog blood samples were collected and sand flies captured. Samples were molecularly tested for *Leishmania* spp. and sandflies for origin of blood meal. Dog sera were tested by IFAT for both *Leishmania* species and reptilian blood and intestine of sand flies were cultured. Four *Tarentola mauritanica* geckoes were positive for *L*. *infantum* or *L*. *tarentolae*; moreover, amastigote-like forms of *L*. *infantum* were observed in the bone marrow. 24/294 sand flies scored PCR positive for *Leishmania* spp. Reptile and dog blood were found in *S*. *minuta*, dog and human in *Ph*. *perniciosus* and dog in *Phlebotomus neglectus*. Two axenic strains of *L*. *tarentolae* were obtained. Twelve of 19 dogs scored positive for *L*. *infantum* and *L*. *tarentolae* by IFAT and three of them also for *L*. *infantum* by PCR, and six by qPCR. Data confirm the sympatric circulation of *L*. *infantum* and *L*. *tarentolae* in geckoes, sand flies, and dogs, and suggest that geckoes may be infected with *L*. *infantum*.

## Introduction

Leishmaniases are important diseases affecting mammals, including humans, in tropical, subtropical, and temperate regions, with more than 350 million people infected worldwide [1]. The genus *Leishmania* (Kinetoplastea, Trypanosomatidae), transmitted predominantly by phlebotomine sand flies (Diptera, Psychodidae), includes more than fifty species which infect mammals and reptiles. From those, about twenty parasitize humans, causing cutaneous, mucocutaneous and visceral leishmaniasis [2,3]. In the Mediterranean basin, *Leishmania infantum* is the main species, causing zoonotic cutaneous and visceral leishmaniasis in humans, and infects more than 2.5 million dogs [4]. In the same area, other pathogenic species of the subgenus *Leishmania* occur (e.g., *Leishmania donovani* in Cyprus and *Leishmania tropica* in Greece), while species typical of reptiles, belonging to the subgenus *Sauroleishmania*, (e.g., *Leishmania chameleonis* and *Leishmania tarentolae*) were found in Algeria, France, and Italy [3,5–7]. Indeed, ecological and anthropic drivers (i.e., climate changes, animal translocation, wildlife movements, or globalization) have amplified the risk of alien *Leishmania* spp. introduction [8], and the spreading of sand fly populations in new localities results in the northward shift of leishmaniasis [9].

The abovementioned factors pose new challenges to medical and veterinary practitioners, given the possibility of diagnostic inaccuracies or cross-reactivities, such as in the case of the simultaneous occurrence of *L*. *infantum* and *L*. *tropica* in dogs in Israel [10] and of *L*. *infantum* and *L*. *tarentolae* in dogs in Italy [7].

In particular, saurian-associated *L*. *tarentolae* has been detected in geckoes *Tarentola mauritanica* and *Mediodactylus kotschyi* from Italy [11] and *Tarentola annularis* from Sudan [12]. Recently, this species was molecularly detected in lizards *Podarcis filfolensis* and *Podarcis siculus* [7,13]. The species was molecularly detected also in human blood in central Italy [14], in islanders of the Pelagie archipelago [15] and in sheltered dogs in Italy [7]. Mammalian species *L*. *infantum* was molecularly detected in lizards (i.e., *P*. *siculus*) and geckoes (i.e., *T*. *mauritanica*, *Hemidactylus turcicus*) inhabiting dog shelters in southern Italy [13]. The high abundance of the natural vector *Sergentomyia minuta* and the detection of human and dog blood in engorged females [14,16] suggest the possibility of mammalian exposure to *L*. *tarentolae*. Indeed, both *L*. *infantum* and *L*. *tarentolae* may infect the same sand fly species where their distribution overlaps. In Italy, this is the case of *Phlebotomus perfiliewi* and *Phlebotomus perniciosus* that were molecularly positive for *L*. *tarentolae* [7,14] and *S*. *minuta* for *L*. *infantum* [14,17–19]. In addition, experimental infections demonstrated that *L*. *tarentolae* develops in *Ph*. *perniciosus* and *Phlebotomus papatasi* [20], the main vectors of *L*. *infantum* and *Leishmania major*, respectively. Although scientific interest on *L*. *tarentolae* is continually growing, its life cycle, pathogenicity, tropism and overall biology remain largely unknown, despite many isolations and molecular characterization efforts [11,20–23]. The obtained strains have been of great importance for the development of biotechnological tools, given by the production of cultured promastigotes at the industrial level [2,24]. In addition, two isolates are available commercially in culture, namely, TarII (ATCC: 30267) (Algeria, 1939; [21]) and LEM125 (France, 1981; [25]) strains, both obtained from *T*. *mauritanica* geckoes. Importantly, LEM125 strains can be transiently infectious to mammals, hence posing a biosafety risk not yet assessed [26–28]. Conversely, TarII strains are considered nonpathogenic and have suffered considerable modifications in their kinetoplast DNA (kDNA) due to the constant culture passages through the many years since the first isolation, losing proteins not essential for their life cycle in culture medium [24]. However, the whole genome sequence is available only for TarII strain [29,30]. Therefore, new information on the epidemiology and biology of *L*. *tarentolae*, as well as the isolation of new strains are needed to better understand the possible infection in mammals. Overall, this study aimed to evaluate the circulation of *L*. *tarentolae* and *L*. *infantum* in reptiles, sand flies, and dogs in an endemic area of canine leishmaniasis (CanL) and to obtain new strains of both *Leishmania* species in axenic cultures.

## Methods

### Ethics statement

Protocols for the collection of reptiles were approved by the ethical committee of the Department of Veterinary Medicine of the University of Bari, Italy (Prot. Uniba 12/20), and authorized by the Ministry for Environment, Land and Sea Protection of Italy (Approval Number 0073267/2019), the Societas Herpetologica Italica and the Istituto Superiore per la Protezione e la Ricerca Ambientale (Approval Number 71216).

**Fig 1:**
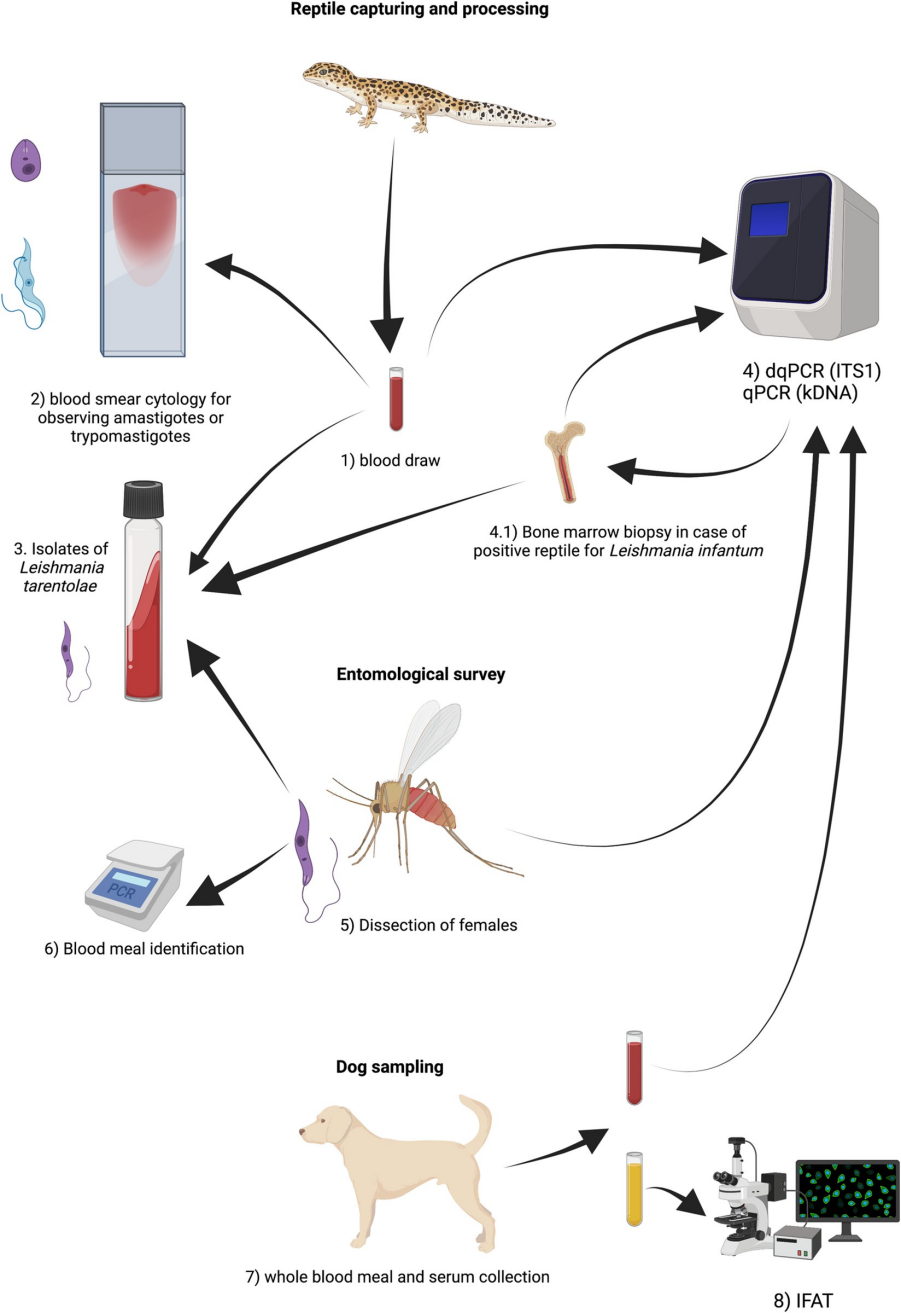
Flowchart of methods and samples used, divided by sections: reptile capturing and processing, entomological survey and dog sampling. Created in: https://biorender.com/.

Methods and samples are summarized in a flowchart (Fig 1).

### Study area

**Fig 2:**
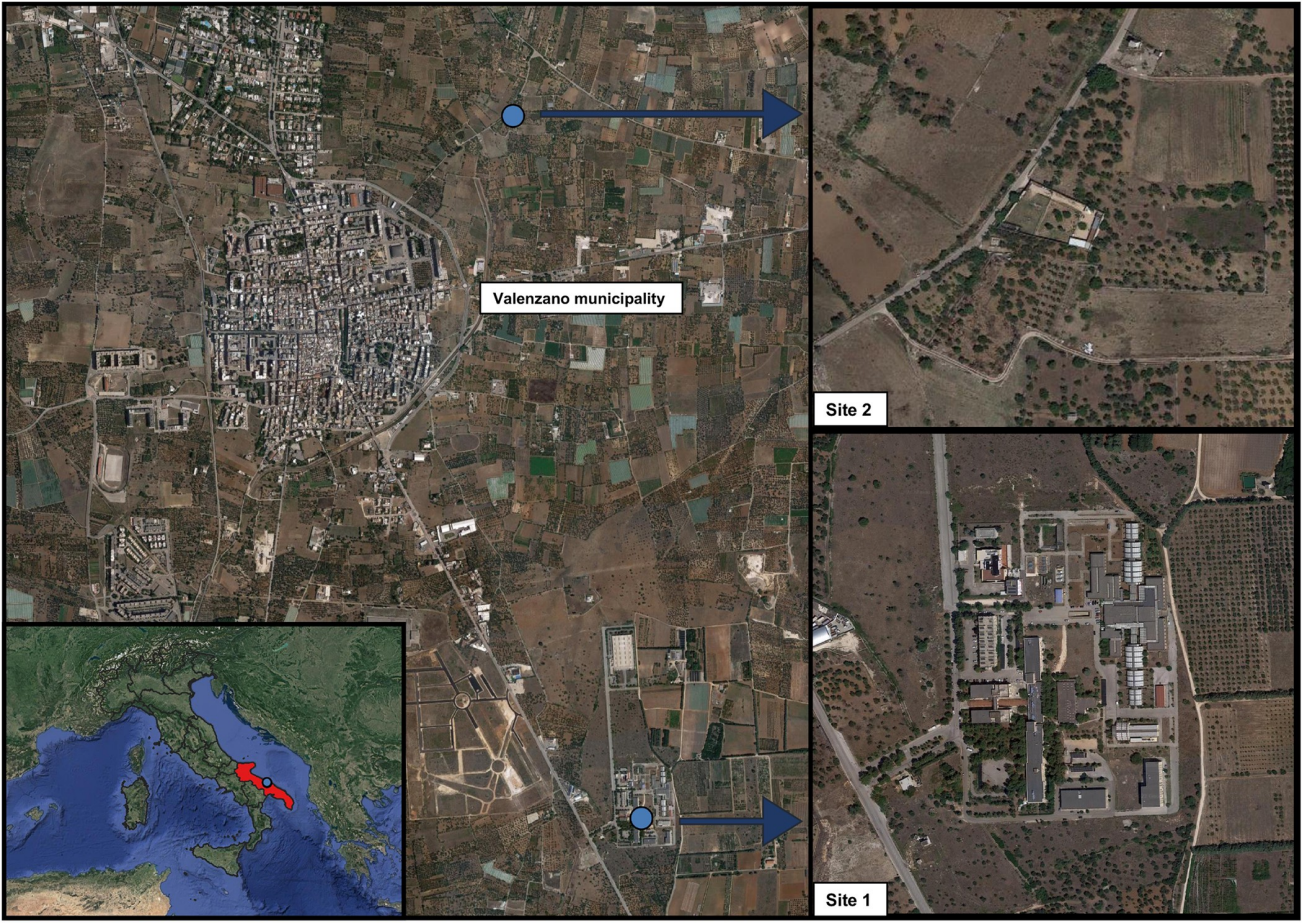
Geographic location of reptile and dog blood sample collection sites and sand fly capturing, in the surroundings of Valenzano municipality, Apulia, Region. (Publicly available satellite shapefiles from http://mt0.google.com/vt/lyrs=s&hl=en&x={x}&y={y}&z={z}; in QGIS 3.4).

**Fig 3:**
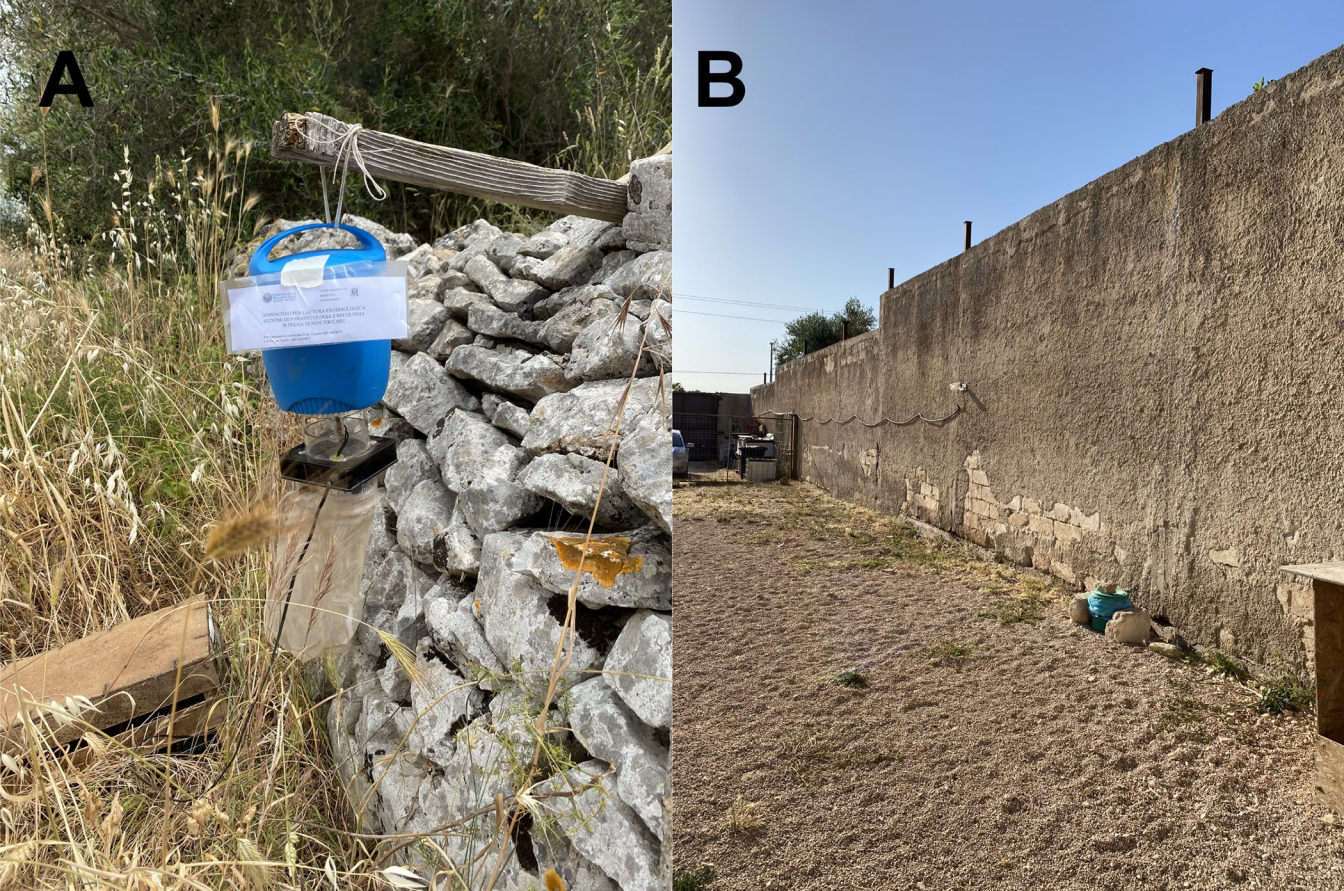
Environmental characteristics of both collection sites. A) “muretto a secco” where the CDC light traps were placed in the campus of Veterinary Medicine, University of Bari “Aldo Moro”. B) a high-walled private-owned dog shelter.

Geckoes, lizards, and sand flies were collected from May to November 2021 in two locations endemic for CanL, near Valenzano municipality (with 4.2 km of distance between each other), Apulia region, Italy [9]. These locations were Campus of Veterinary Medicine, University of Bari “Aldo Moro” (site 1; 41°1’31.584"N, 16°54’3.6288"E) and a private owned dog shelter (site 2; 41°03’04.3"N, 16°53’39.7"E), where dog blood samples were also collected and screened (Fig 2). Site 1 had a Mediterranean environment characterized by olive trees, the presence of typical “*muretti a secco*” (stone walls) where reptiles and sand flies thrive (Fig 3A), while site 2 was a high-walled shelter, with few olive trees where dogs and pigeons were kept (Fig 3B).

### Sample collection

#### Reptile capturing and processing

Adult reptiles (except for gravid females) were captured by lassoing or by hand, or in the case of snakes using herpetological hooks and sampled before release in the original home range. A small amount of blood (~200μl) obtained via cardiocentesis (in snakes from the ventral coccygeal vein) was used for smears (stained with Diff-Quik), cultivation (~50μl) in a modified Tobie-Evans (TE) medium [31], and the rest was stored at −20°C until molecularly processed. Geckoes, positive for *L*. *infantum* in blood, were further assessed by bone-marrow biopsy of the femur, according to protocols described elsewhere [32,33]. Briefly, a regular hypodermic needle of adequate size and length (30G X 5/16”) was used to collect the sample. The area was prepared aseptically, and the needle was inserted into the bone with continuous and steady pressure and a slight rotational movement. The bone marrow was collected by aspirating the syringe with a slight but steady negative pressure. The obtained material was used for smears in films, cultivation, and DNA extraction.

#### Entomological survey

During the study period, sand flies were collected twice a week using a sticky trap area of 1 m^2^ (32 papers; 21.0 × 29.7 cm) and 4 CDC light traps set from 5:00 p.m. to 8:00 a.m. Collections were carried out during the sand fly activity season [13] until the total absence of sand flies (i.e., three consecutive negative captures). Specimens were stored in 70% ethanol and morphologically identified using taxonomic keys [34]. Alive females collected by CDC traps were dissected with a drop of saline solution and the gut was observed under a microscope to determine the presence of flagellates [20]. The gut content of positive females was cultivated in a modified Tobie-Evans (TE) medium [31].

#### Dog sampling

Nineteen dogs housed in site 2 were sampled in May and November 2021. A complete physical examination was performed by a veterinarian to assess the health status of the animals. From each dog, whole blood was collected in vacuum containers with K3 EDTA (2.5 ml) and serum collection tubes with clot activator (5 ml).

### Serological testing

Dog serum samples were tested by IFAT for the detection of IgG anti-*L*. *infantum* as described previously [35]. To evaluate exposure to *L*. *tarentolae*, IFAT was performed using promastigotes of *L*. *tarentolae* (strain RTAR/IT/81/ISS21-G.6c/LEM124) following the same procedure as for *L*. *infantum* IFAT. For both IFAT, serum samples of a dog positive for *L*. *infantum* by cytological and molecular analyses, and a healthy dog negative for *L*. *infantum*, were used as positive and negative controls, respectively. The samples were scored as positive when they produced clear cytoplasmic and membrane fluorescence of promastigotes from a cut-off dilution of 1:80 [36]. Positive sera were titrated by serial dilutions until negative results were obtained.

### Molecular biology

Genomic DNA (gDNA) was extracted from reptile and dog blood samples and cultures, using the GenUPBlood DNA commercial kit (Biotechrabbit GmbH, Hennigsdorf, Germany), according to the manufacturer’s instructions. DNA was extracted from bone marrow films using the QIAamp DNA Micro Kit (QIAGEN, Hilden, Germany). gDNA was extracted from the thorax and abdomen (heads and last abdominal segments were removed for morphological identification) of each sand fly female (*n*  =  294) using an in-house method as previously described [37]. All samples (i.e., blood, bone marrow, and sand flies) were tested by duplex real-time PCR (dqPCR) for the detection of *L*. *infantum* and *L*. *tarentolae* and were considered positive with Ct values up to 38.0 and 38.6, respectively, as described previously [38]. Blood samples were also tested for *L*. *infantum* kDNA minicircle (120 bp) by real-time PCR (qPCR), using the protocol described elsewhere [39]. gDNA from *L*. *infantum* promastigotes cultured in Tobie-Evans medium from an infected dog living in Italy (zymodeme MON-1) and from *L*. *tarentolae* (strain RTAR/IT/81/ISS21-G.6c/LEM124) were used as controls. gDNA extracted from a blood sample from a lizard and a dog tested negative for *Leishmania* spp. was used as a negative control.

For sequence analyses, culture isolates were amplified by conventional PCR (cPCR) using primers L5.8S/LITSR targeting a partial region of the internal transcribed spacer 1 (ITS1, ~300bp) and ran the PCR protocol as described elsewhere [40]. A fragment of the Heat-shock protein 70 gene (*hsp*70, 1,245 pb) was also amplified using specific primers and the PCR protocol was run as described elsewhere [41].

Engorged sand flies (*n*  =  10) were tested for blood-meal identification by cPCR using primers targeting the vertebrate 16S rRNA gene (600 bp), and the PCR protocol was run as previously described [14]. All PCR reactions consisted of 4 μl of gDNA and 46 μl of PCR mix containing 3 mM MgCl2, 10 mM Tris–HCl (pH 8.3) and 50 mM KCl, 125 μM of each dNTP, 1 pmol/μl of each primer, and 2 U of AmpliTaq Gold (Applied Biosystems, Foster City, CA, USA). The amplified products were examined on 2% agarose gels stained with GelRed (VWR International PBI, Milan, Italy) and visualized on a Gel Logic 100 gel documentation system (Kodak, NY, USA). The amplicons were purified and sequenced in both directions using the same primers as for PCR, employing Big Dye Terminator v.3.1 chemistry in an automated sequencer (3130 Genetic Analyzer, Applied Biosystems, Foster City, CA, USA). All sequences were aligned using Geneious prime software and compared to those available in GenBank using the BLASTn tool (http://blast.ncbi.nlm.nih.gov/blast.cgi).

The genetic relationship of *Leishmania* species was evaluated using representative *hsp*70 sequences obtained from culture isolates, and those of reference laboratory strains of *L*. *tarentolae* and *L*. *infantum*, along with relevant trypanosomatids available in the GenBank database. The phylogenetic tree was inferred using the maximum likelihood (ML) method based on the Kimura 3-parameter model [42], selected by best-fit model analysis, and based on the lowest score obtained by Bayesian Information Criterion (BCI), using MEGA6 software (Kimura, 1980). A discrete Gamma distribution was used to model evolutionary rate differences between sites (4 categories (+G, parameter = 0.1772)). Evolutionary analyses were conducted with 5000 bootstrap replicates using MEGA6 software [43]. The corresponding *hsp*70 sequence of *Trypanosoma brucei gambiense (*Accession number: KP208736.1) was used as an outgroup.

## Results

### Reptile capturing and processing

**Fig 4:**
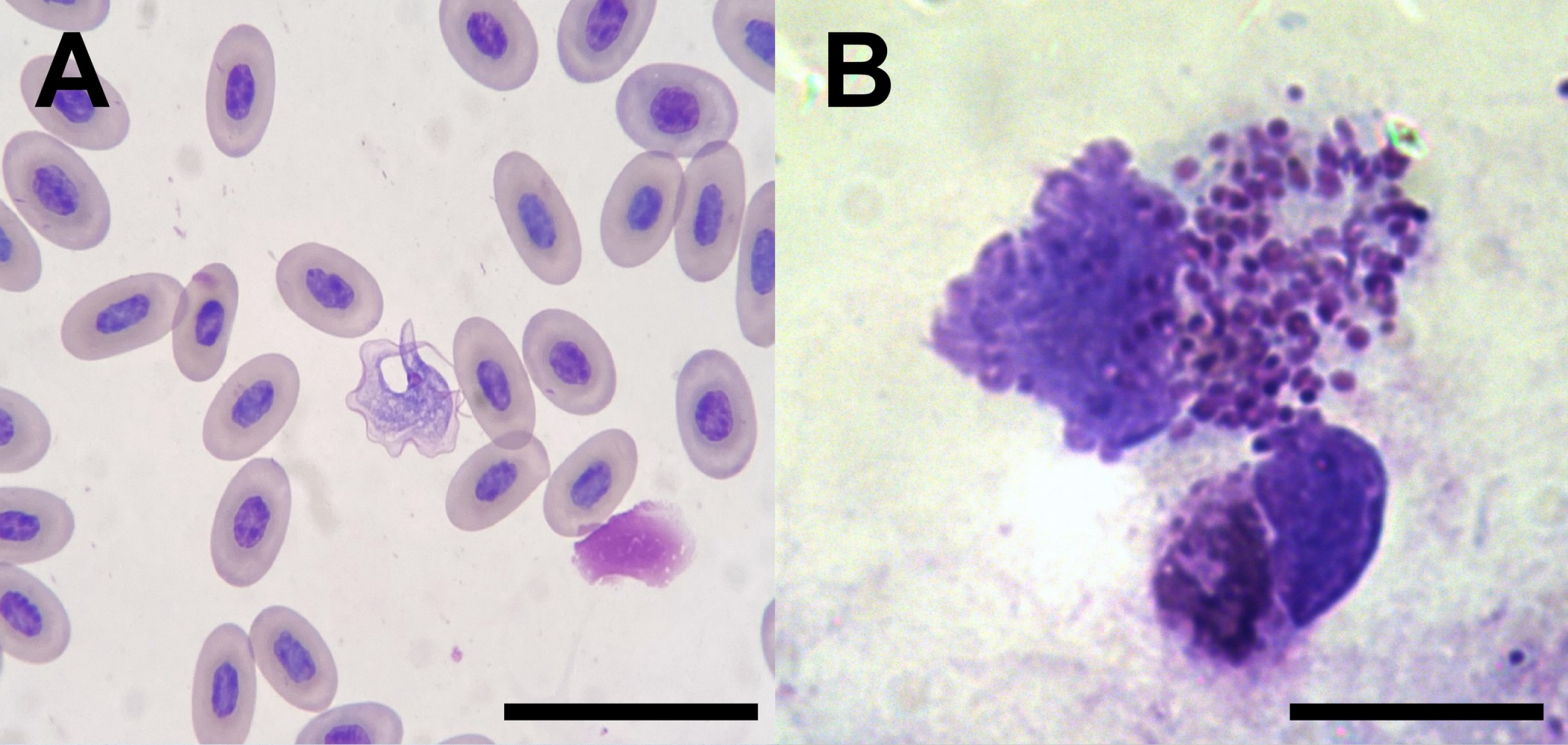
Trypanosomatidae from geckoes. A) *Trypanosoma* sp. trypomastigotes in blood of *Tarentola mauritanica* gecko. B) molecularly identified *Leishmania infantum* amastigote-like forms in leukocyte of *Tarentola mauritanica* gecko. Scale bars A) 50 μm; B) 10 μm.

During the seven-month study period, 37 reptiles of three species (two *Hierophis carbonarius* snakes, seven *Podarcis siculus* lizards, and 28 *T*. *mauritanica* geckoes) were captured and sampled. In particular, two *H*. *carbonarius*, seven *P*. *siculus*, 13 *T*. *mauritanica*) were collected at site 1, while 15 *T*. *mauritanica* were collected at site 2. *Leishmania tarentolae* was isolated from a *T*. *mauritanica* gecko from site 2 (Table 1). On cytological blood smear examination, trypomastigotes, most likely of *Trypanosoma* cf. *platydactyli*, (Fig 4A) were observed in three *T*. *mauritanica* geckoes from site 2, whereas amastigote forms of *Leishmania* spp. were not found in blood. Of the 37 reptile blood samples examined by dqPCR and qPCR, four (10.81%) *T*. *mauritanica* geckoes were positive at site 2. Specifically, two geckoes were positive for *L*. *tarentolae*, one being the source of the *L*. *tarentolae* strain RTAR/IT/21/Ct-25.09, while two other geckoes were positive for *L*. *infantum* (one scored positive by dqPCR and one by qPCR; Table 1). Bone marrow films from the two *L*. *infantum* PCR positive geckoes revealed amastigote forms within monocytes and macrophages (Fig 4B; Table 1). Furthermore, DNA extracted from bone marrow films was positive in one gecko (dqPCR Ct-38.6, qPCR Ct-29.07), confirming *L*. *infantum* infection (Table 1).

**Table 1:** Trypanosomatid detection in *Tarentola mauritanica* geckoes using blood cytology, isolation (in TE medium), dqPCR and qPCR; values of the threshold cycle (Ct) are reported.

Gecko identification number	Blood cytology	Isolation	dqPCR (Ct)	qPCR (Ct)
3	*Leishmania* spp.*	-	*Leishmania infantum* (37.50–38.6*)	*Leishmania infantum* (29.07*)
5	*Trypanosoma* sp. *Leishmania* spp.*	-	-	*Leishmania infantum* (36.81)
6	*Trypanosoma* sp.	*Leishmania tarentolae*	*Leishmania tarentolae* (26.95)	-
14	*Trypanosoma* sp.	-	-	-
15	-	-	*Leishmania tarentolae* (25.09)	-

*from bone marrow.

### Entomological survey

A total of 716 phlebotomine sand flies (i.e., 474 *S*. *minuta*, 206 *Ph*. *perniciosus*, and 36 *Ph*. *neglectus*) were collected, of which 294 were females (231 *S*. *minuta*, 52 *Ph*. *perniciosus*, and 11 *Ph*. *neglectus*). Of the sand fly females, 24 scored positive for *Leishmania* spp. (8.1%) by dqPCR (Table 2). Among them, 21 (87.5%) *S*. *minuta* and two (8.3%) *Ph*. *perniciosus* were positive for *L*. *tarentolae*, while one (4.2%) *Ph*. *perniciosus* for *L*. *infantum*. Out of the 44 females collected using CDC light traps, four *S*. *minuta* were microscopically positive for promastigotes; *L*. *tarentolae* was confirmed by dqPCR for three of them only and one axenic culture was established after 30 days. Furthermore, host blood meal sequences were obtained for five out of ten engorged females, with high nucleotide identity (99.8–100%) of *T*. *mauritanica* (1×; AN JQ425060) and dog (1×; AN MN699634) in *S*. *minuta*, of humans (2×; AN OL521838 and MK617278) in *Ph*. *perniciosus*, and dog (1×; AN MN699634) in *Ph*. *neglectus*.

**Table 2:** Sand fly females tested for *Leishmania infantum* and/or *Leishmania tarentolae* by duplex quantitative PCR. The mean (M), minimum (Min), maximum (Max) and standard deviation (sd) values of the threshold cycle (Ct) are reported.

Sand flies	*Leishmania tarentolae*				*Leishmania infantum*				
	P/T (%)		Ct		P/T (%)		Ct		P/T (%)
		M	Min-max	sd		M	Min-max	sd	
*S*. *minuta*	21/231 (9.1)	33.35	19.3–37.73	6.3	0/231	-	-	-	21/231 (9.1)
*Ph*. *perniciosus*	2/52 (3.8)	37.58	36.54–38	1	1/52 (2)	29.69	-	-	3/52 (5.7)
*Ph*. *neglectus*	0/11	-	-	-	0/11	-	-	-	0/11
P/T (%)	23/294 (7.8)				1/294 (0.3)				24/294 (8.1)

### Dog sampling

Of 19 dogs serologically examined, 12 (63.2%) scored positive against promastigotes of *L*. *infantum* and *L*. *tarentolae* by IFAT in May and 11 (58.8%) in November 2021 (Table 3). Dog blood samples tested by dqPCR and qPCR only yielded positive results for *L*. *infantum* in November 2021. Specifically, three dog samples were positive by dqPCR and six samples by qPCR, being three animals positive for both methods (Table 3).

**Table 3:** Antibody titers against *Leishmania infantum* and *Leishmania tarentolae* promastigotes detected by indirect fluorescent antibody test (IFAT) according to sampling time (May and November 2021) and serum dilution (1:80 to 1:2560). Values of the threshold cycle (Ct) are reported for dqPCR and qPCR that were only positive for *Leishmania infantum*.

Dog No.	May 2021		November 2021			
	IFAT		IFAT		dqPCR (Ct)	qPCR (Ct)
	*Leishmania infantum*	*Leishmania tarentolae*	*Leishmania infantum*	*Leishmania tarentolae*	*Leishmania infantum*	
**1**	1:2560	1:2560	1:2560	1:1280	33.80	26.20
**2**	1:320	1:320	1:640	1:640	35.23	29.18
**3**	1:80	1:80	Died		-	-
**4**	1:320	1:640	1:640	1:640	-	-
**5**	1:320	1:320	1:640	1:160	-	-
**6**	1:80	1:80	1:160	-	-	-
**7**	1:640	1:640	1:2560	1:1280	-	27.93
**8**	1:640	1:320	1:640	1:320	-	-
**9**	-	-	1:80	1:80	-	29.06
**10**	1:2560	1:2560	1:2560	1:640	-	-
**11**	1:1280	1:1280	1:2560	1:2560	38.34	28.05
**12**	1:640	1:1280	1:1280	1:640	-	32.16
**13**	-	-	-	-	-	-
**14**	-	-	-	-	-	-
**15**	1:80	1:80	-	-	-	-
**16**	-	-	-	-	-	-
**17**	-	-	-	-	-	-
**18**	-	-	-	-	-	-
**19**	-	-	-	-	-	-

### Isolation and sequence analyses

**Fig 5:**
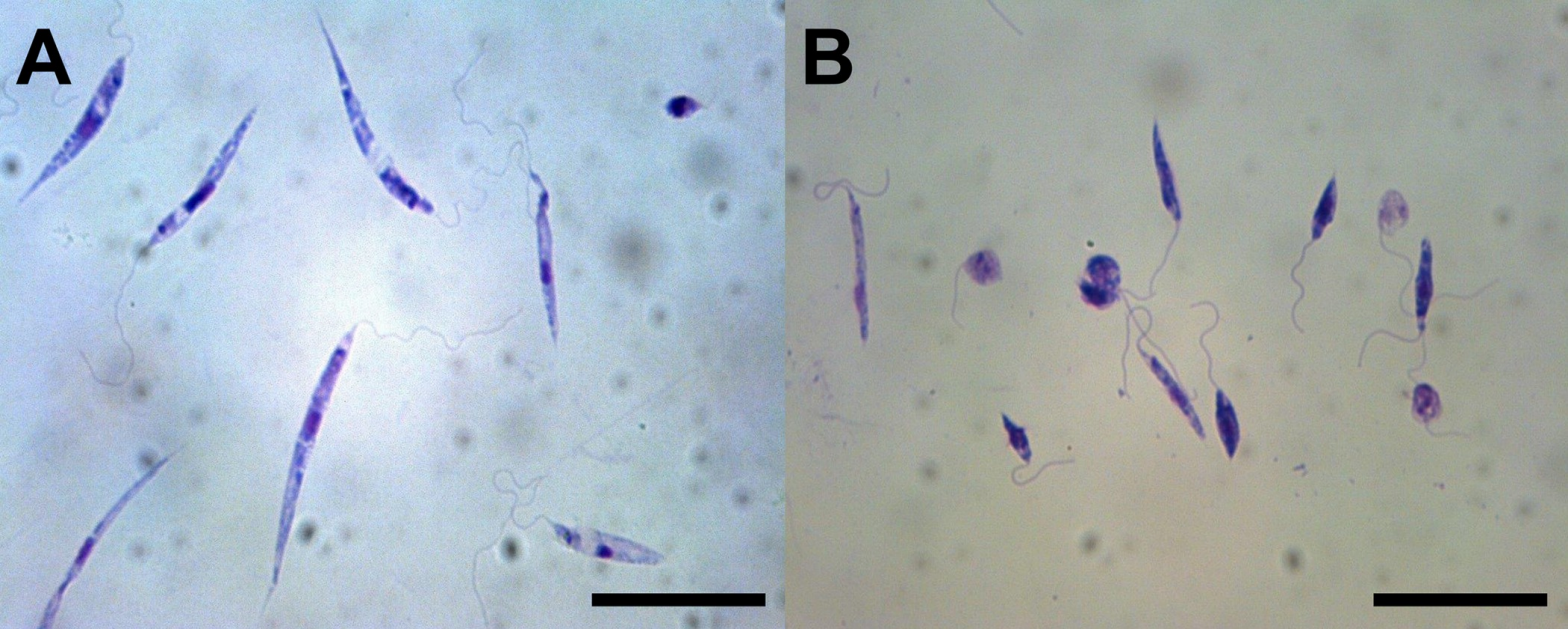
Cultured promastigotes of isolated strains. A) the strain RTAR/IT/21/RI325 isolated from a gecko *Tarentola mauritanica*, with longer body and flagella. B) the strain ISER/IT/21/SF178 isolated from a sand fly female *Sergentomyia minuta*. Scale bars A,B) 30 μm.

**Fig 6:**
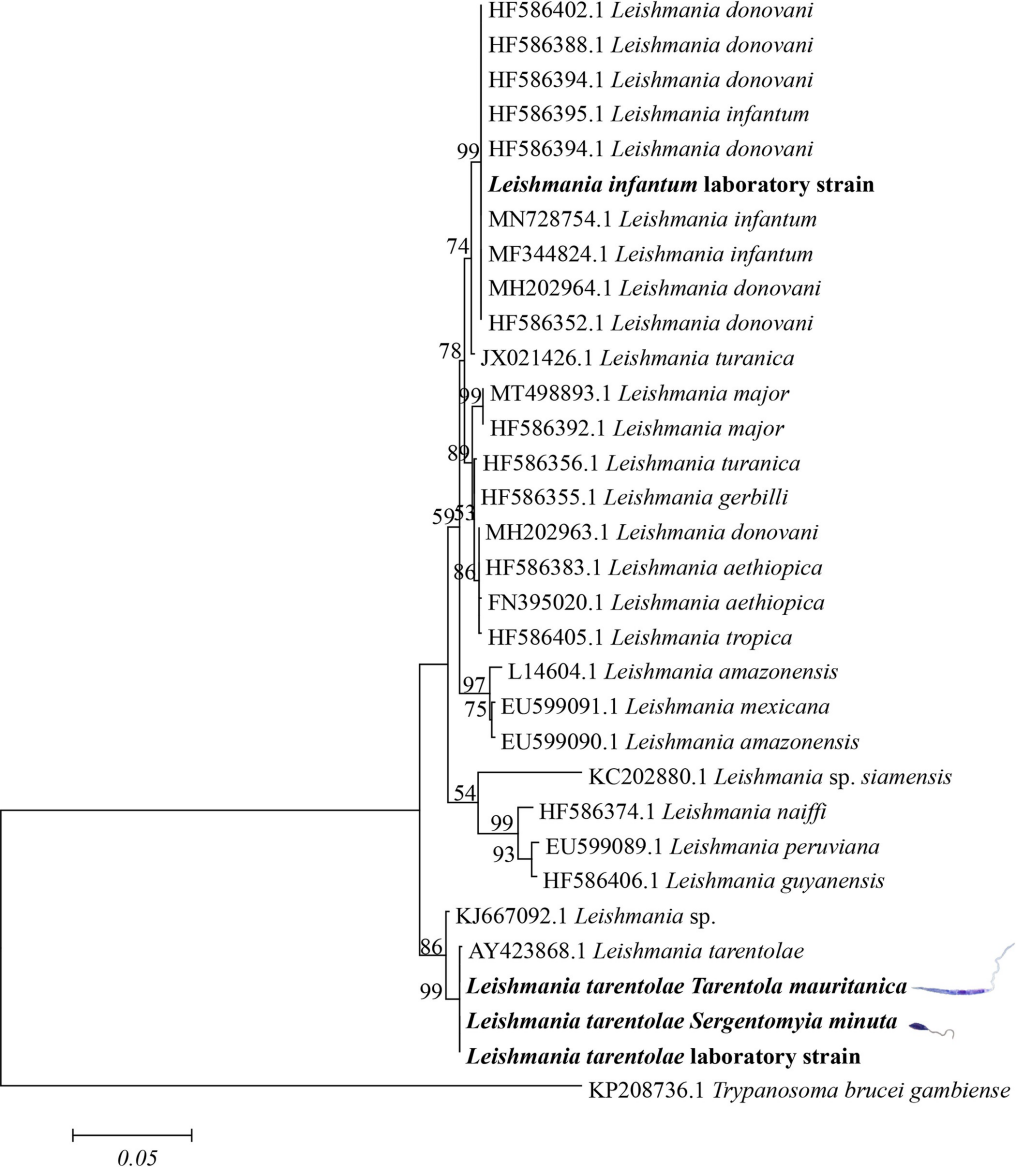
Phylogenetic tree based on *Leishmania hsp*70 sequences inferred using the maximum likelihood method based on the Kimura 3-parameter model. *Trypanosoma brucei gambiense* was used as an outgroup. Scale bar indicates nucleotide substitution per site. The sequences of *Leishmania* spp. obtained in this study are in bold.

Overall, new strains of *L*. *tarentolae* were isolated from *T*. *mauritanica* (strain RTAR/IT/21/RI325) (Fig 5A) and from *S*. *minuta* (ISER/IT/21/SF178) (Fig 5B) from the same locality (site 2). Sequence analyses of the ITS1 region and *hsp*70 gene (Fig 6) confirmed the *L*. *tarentolae* identification of the isolates (Accession Numbers: RTAR/IT/21/RI325 –OM831140; ISER/IT/21/SF178 –OM831137).

## Discussion

Data demonstrate the sympatric circulation of *L*. *infantum* and *L*. *tarentolae* in geckoes, sand flies, and dogs in an area endemic for CanL. Synanthropic geckoes were found to be PCR positive for *L*. *infantum* and *Ph*. *perniciosus* for *L*. *tarentolae*. Importantly, the finding of *L*. *infantum* amastigotes in the bone marrow of geckoes suggests that these animals are not only exposed, but also infected with this *Leishmania* species.

Both reptile species (i.e., *P*. *siculus*, *T*. *mauritanica*) sampled in this study, have previously been recorded PCR positive to *L*. *infantum* and *L*. *tarentolae* in the same geographical area, though the prevalence for both *L*. *tarentolae* (12.5%) and *L*. *infantum* (4.1%) was higher [13]. In the study presented herein, trypanosomatids were only detected in *T*. *mauritanica* geckoes probably because it was the main species of reptiles collected. Importantly, sympatric occurrence of these protists (with no co-infection of both *Leishmania* spp.) was observed in geckoes collected from site 2 (a dog shelter), with a prevalence of 13.3% for both *Leishmania* spp. and 26.6% for trypomastigotes. *Trypanosoma* sp. was observed in this study co-infecting three hosts with *L*. *tarentolae*, in blood cytology, with different combinations of diagnostic techniques. Though not identified at species level, *Trypanosoma* cf. *platydactyli* was previously described (prevalence of 27.5%) co-infecting with *L*. *tarentolae*, in *T*. *mauritanica* geckoes captured in the surroundings of the Monopoli municipality [31], a city located in the same region (i.e., Apulia) of the present study sites, merely 40 km away. This trypanosomatid has posed an unexpected hindrance for the morphological study of *Leishmania* spp. in Mediterranean reptiles, being once synonymized with *L*. *tarentolae* as their developing forms in culture can be confused (i.e., trypomastigotes may develop into promastigotes after some passages; [22]). Although *L*. *infantum* has already been molecularly detected in reptiles [13], this is the first time that amastigote-like forms of this mammalian-associated *Leishmania* species have been observed in bone marrow aspirate by cytology and further confirmed molecularly. Therefore, these data suggest that reptiles may be infected with *L*. *infantum*, further supporting previous studies in which *Leishmania* spp. pathogenic to mammals were molecularly detected in reptiles [44–46]. Accordingly, efforts should be made to isolate *L*. *infantum* from reptiles to confirm their role as reservoirs of this medically and veterinary important protist.

The detected species composition and abundance of sand flies are in agreement with previous studies in endemic CanL areas [13,14,16,18], where *S*. *minuta* was the most abundant species, followed by *Ph*. *perniciosus*, the natural vector of *L*. *infantum*. The isolation of *Leishmania* parasites from sand flies confirmed the vector competence of *S*. *minuta* for *L*. *tarentolae* and represents a new wild type strain for this species [11,31]. Importantly, molecular positivity of *S*. *minuta* for *L*. *infantum* [13,18,19] and positivity for dog DNA in blood meal [16] may imply a putative role as a vector for this species. However, experimental studies are needed to verify the vector capacity of *S*. *minuta* for *L*. *infantum*.

The serological results in the studied canine population were consistent with the expected epidemiological scenario, also determined by the molecular detection of only *L*. *infantum* in dog blood. Moreover, *L*. *infantum* positive blood samples corresponded to animals that had antibody titers higher than 1:640, with lower Ct values in qPCR, which agrees with the higher sensitivity of minicircle qPCR [39]. Indeed, the usefulness of blood qPCR to detect *Leishmania* is correlated with antibody titration [47], and as high positive predictive value based on clinical evaluation [48]. Nonetheless, given the limited group of dogs selected, discarding a co-infection or a previous exposure to *L*. *tarentolae* is difficult. However, this study warrants the high possibility of cross-reactivity seen in most cases of sympatric occurrence of trypanosomatids using IFAT methods [49,50]. Thus, to distinguish the infecting species of *Leishmania*, specific confirmatory serological tests should be developed.

This study also represents the most recent isolation of *L*. *tarentolae* strains from both reptiles and sand flies, thus not suffering from genetic drift due to the long-term cultivation. Although the ITS1 and *hsp70* sequences were highly similar to those of the reference strains, such new strains may represent transiently infectious parasites that should be further molecularly characterized and subjected to phagocytosis and *in vitro* infectivity analyses [27,29]. Indeed, further attempts should be made to isolate *L*. *tarentolae* from other hosts (i.e., dogs and humans) to fully unravel the infection in mammalian hosts.

The epidemiological scenarios of canine leishmaniasis in the Mediterranean basin can change depending on multiple factors, such as geographic area, sand fly species, and/or canine populations. The scenario observed in the periurban areas of southern Italy adds to this factor the presence of often ignored sand fly, reptile, and *Leishmania* species, namely *S*. *minuta*, *T*. *mauritanica*, and *L*. *tarentolae*. Synanthropic omnipresent geckoes are exposed not only to their specific *Leishmania* species, but also to *L*. *infantum*, which can develop into amastigotes in the bone marrow. The studied sand fly species display different levels of anthropophilic feeding behavior. The isolation of new *L*. *tarentolae* strains may provide new information on transient infection, similar to the LEM125 strains. Confirming the isolation of *L*. *infantum* from reptiles and *L*. *tarentolae* from mammals is needed to unravel the epidemiological context given by the sympatric occurrence of both *Leishmania* species.

## References

[1] World Health Organization (WHO). Leishmaniasis [cited 2022 March 25]. Available from: https://www.who.int/news-room/fact-sheets/detail/leishmaniasis

[2] Cantacessi C, Dantas-Torres F, Nolan MJ, Otranto D. The past, present, and future of *Leishmania* genomics and transcriptomics. Trends Parasitol. 2015;31: 100–108. doi: 10.1016/j.pt.2014.12.012 25638444 PMC4356521

[3] Akhoundi M, Kuhls K, Cannet A, Votýpka J, Marty P, Delaunay P, et al. A historical overview of the classification, evolution, and dispersion of *Leishmania* parasites and sandflies. PLoS Negl Trop Dis. 2016;10: e0004349. doi: 10.1371/journal.pntd.0004349 26937644 PMC4777430

[4] Berriatua E, Maia C, Conceição C, Özbel Y, Töz S, Baneth G, et al. Leishmaniases in the European Union and Neighboring Countries. Emerg Infect Dis. 2021;27: 1723–1727. doi: 10.3201/eid2706.210239 34013857 PMC8153892

[5] Léger N, Depaquit J. *Leishmania donovani* leishmaniasis in Cyprus. Lancet Infect Dis. 2008; 8:402. doi: 10.1016/S1473-3099(08)70132-4 18582830

[6] Ntais P, Sifaki-Pistola D, Christodoulou V, Messaritakis I, Pratlong F, Poupalos G, et al. Leishmaniases in Greece. Am J Trop Med Hyg. 2013;89: 906–915. doi: 10.4269/ajtmh.13-0070 24062479 PMC3820334

[7] Mendoza-Roldan JA, Latrofa MS, Iatta R, Manoj RRS, Panarese R, Annoscia G, et al. Detection of *Leishmania tarentolae* in lizards, sand flies and dogs in southern Italy, where *Leishmania infantum* is endemic: hindrances and opportunities. Parasit Vectors. 2021;14: 1–12. 34493323 10.1186/s13071-021-04973-2 PMC8423600

[8] Colwell DD, Dantas-Torres F, Otranto D. Vector-borne parasitic zoonoses: emerging scenarios and new perspectives. Vet Parasitol. 2011;182: 14–21. doi: 10.1016/j.vetpar.2011.07.012 21852040

[9] Mendoza-Roldan J, Benelli G, Panarese R, Iatta R, Furlanello T, Beugnet F, et al. *Leishmania infantum* and *Dirofilaria immitis* infections in Italy, 2009–2019: changing distribution patterns. Parasit Vectors. 2020;13: 1–8. 32293524 10.1186/s13071-020-04063-9 PMC7161282

[10] Baneth G, Zivotofsky D, Nachum-Biala Y, Yasur-Landau D, Botero AM. Mucocutaneous *Leishmania tropica* infection in a dog from a human cutaneous leishmaniasis focus. Parasit Vectors. 2014;7: 1–5. 24661746 10.1186/1756-3305-7-118 PMC3987837

[11] Pozio E, Gramiccia M, Gradoni L, Maroli M. Hemoflagellates in *Cyrtodactylus kotschyi* (Steindachner, 1870) (Reptilia, Gekkonidae) in Italy. Acta Trop. 1983;40: 399–400. 6142639

[12] Elwasila M. *Leishmania tarentolae* Wenyon, 1921 from the gecko *Tarentola annularis* in the Sudan. Parasitol Res. 1988;74: 591–592. doi: 10.1007/BF00531640 3194372

[13] Mendoza-Roldan JA, Latrofa MS, Tarallo VD, Manoj RR, Bezerra-Santos MA, Annoscia G, et al. *Leishmania* spp. in Squamata reptiles from the Mediterranean basin. Transbound Emerg Dis. 2022: 1–11. 34951929 10.1111/tbed.14438

[14] Pombi M, Giacomi A, Barlozzari G, Mendoza-Roldan J, Macrì G, Otranto D, et al. Molecular detection of *Leishmania* (*Sauroleishmania*) *tarentolae* in human blood and *Leishmania* (*Leishmania*) *infantum* in *Sergentomyia minuta*: unexpected host-parasite contacts. Med Vet Entomol. 2020;34: 470–475. doi: 10.1111/mve.12464 32710462

[15] Iatta R, Mendoza-Roldan JA, Latrofa MS, Cascio A, Brianti E, Pombi M, et al. *Leishmania tarentolae* and *Leishmania infantum* in humans, dogs and cats in the Pelagie archipelago, southern Italy. PLoS Negl Trop Dis. 2021;15: e0009817. doi: 10.1371/journal.pntd.0009817 34555036 PMC8491888

[16] Abbate JM, Maia C, Pereira A, Arfuso F, Gaglio G, Rizzo M, et al. Identification of trypanosomatids and blood feeding preferences of phlebotomine sand fly species common in Sicily, Southern Italy. PLoS One. 2020;15: e0229536. doi: 10.1371/journal.pone.0229536 32155171 PMC7064173

[17] Tarallo VD, Dantas-Torres F, Lia RP, Otranto D. Phlebotomine sand fly population dynamics in a leishmaniasis endemic peri-urban area in southern Italy. Acta Trop. 2010;116: 227–234. doi: 10.1016/j.actatropica.2010.08.013 20816927

[18] Latrofa MS, Iatta R, Dantas-Torres F, Annoscia G, Gabrielli S, Pombi M, et al. Detection of *Leishmania infantum* DNA in phlebotomine sand flies from an area where canine leishmaniosis is endemic in southern Italy. Vet Parasitol. 2018;253: 39–42. doi: 10.1016/j.vetpar.2018.02.006 29605001

[19] Iatta R, Zatelli A, Laricchiuta P, Legrottaglie M, Modry D, Dantas-Torres F, et al. *Leishmania infantum* in tigers and sand flies from a leishmaniasis-endemic area, Southern Italy. Emerg Infect Dis. 2020;26: 1311–1314. doi: 10.3201/eid2606.191668 32441622 PMC7258470

[20] Ticha L, Kykalova B, Sadlova J, Gramiccia M, Gradoni L, Volf P. Development of various *Leishmania* (*Sauroleishmania*) *tarentolae* strains in three *Phlebotomus* species. Microorganisms. 2021;9: 2256. doi: 10.3390/microorganisms9112256 34835382 PMC8622532

[21] Parrot L, Foley H. Sur la frequence de la leishmaniose du gecko dans le Sud oranais. Arch Inst Pasteur Alger, 1939;1: 231–232.

[22] Wallbanks KR, Maazoun R, Canning EU, Rioux JA. The identity of *Leishmania tarentolae* Wenyon 1921. Parasitology. 1985;90: 67–78. doi: 10.1017/s0031182000049027 3982855

[23] Gomez-Eichelmann MC, Holz G Jr, Beach D, Simpson AM, Simpson L. Comparison of several lizard *Leishmania* species and strains in terms of kinetoplast minicircle and maxicircle DNA sequences, nuclear chromosomes, and membrane lipids. Mol Biochem Parasitol. 1988;27: 143–158. doi: 10.1016/0166-6851(88)90034-5 3344003

[24] Klatt S, Simpson L, Maslov DA, Konthur Z. *Leishmania tarentolae*: Taxonomic classification and its application as a promising biotechnological expression host. PLoS Negl Trop Dis. 2019;13: e0007424. doi: 10.1371/journal.pntd.0007424 31344033 PMC6657821

[25] Gao G, Kapushoc ST, Simpson AM, Thiemann OH, Simpson L. Guide RNAs of the recently isolated LEM125 strain of *Leishmania tarentolae*: an unexpected complexity. RNA. 2001;7: 1335–1347. doi: 10.1017/s1355838201018076 11565754 PMC1370176

[26] Simpson L, Holz G Jr. The status of *Leishmania tarentolae*/*Trypanosoma platydactyli*. Parasitol Today. 1988;4: 115–118. doi: 10.1016/0169-4758(88)90043-9 15463063

[27] Taylor VM, Muñoz DL, Cedeño DL, Vélez ID, Jones MA, Robledo SM. *Leishmania tarentolae*: utility as an in vitro model for screening of antileishmanial agents. Exp Parasitol. 2010;126: 471–475. doi: 10.1016/j.exppara.2010.05.016 20685203

[28] Simpson L, Douglass SM, Lake JA, Pellegrini M, Li F. Comparison of the mitochondrial genomes and steady state transcriptomes of two strains of the trypanosomatid parasite, *Leishmania tarentolae*. PLoS Negl Trop Dis. 2015;9: e0003841. doi: 10.1371/journal.pntd.0003841 26204118 PMC4512693

[29] Raymond F, Boisvert S, Roy G, Ritt JF, Légaré D, Isnard A, et al. Genome sequencing of the lizard parasite *Leishmania tarentolae* reveals loss of genes associated to the intracellular stage of human pathogenic species. Nucleic Acids Res. 2012;40: 1131–1147. doi: 10.1093/nar/gkr834 21998295 PMC3273817

[30] Goto Y, Kuroki A, Suzuki K, Yamagishi J. Draft genome sequence of *Leishmania tarentolae* Parrot Tar II, obtained by Single-Molecule Real-Time Sequencing. Microbiol Resour Announc. 2020;9: e00050–20. doi: 10.1128/MRA.00050-20 32439660 PMC7242662

[31] Pozio E, Gramiccia M, Gradoni L, Maroli M. Hémoflagellés de *Tarentola mauritanica* L., 1758 (Reptilia, Gekkonidae). *Leishmania*. *Taxonomie et Phylogenese*. *Montepellier*: *IMEEE*, 149–155.

[32] Redrobe S, MacDonald J. Sample collection and clinical pathology of reptiles. Vet Clin North Am Exot Anim Pract. 1999;2: 709–730. doi: 10.1016/s1094-9194(17)30118-4 11229051

[33] Saggese MD. Clinical approach to the anemic reptile. J Exot Pet Med. 2009;18: 98–111.

[34] Dantas-Torres F, Tarallo VD, Otranto D. Morphological keys for the identification of Italian phlebotomine sand flies (Diptera: Psychodidae: Phlebotominae). Parasit Vectors. 2014;7: 1–6. 25323537 10.1186/s13071-014-0479-5 PMC4203899

[35] Otranto D, Testini G, Dantas-Torres F, Latrofa MS, Diniz PP, Caprariis D, et al. Diagnosis of canine vector-borne diseases in young dogs: a longitudinal study. J Clin Microbiol. 2010;48: 3316–3324. doi: 10.1128/JCM.00379-10 20660218 PMC2937705

[36] Otranto D, Paradies P, Caprariis D, Stanneck D, Testini G, Grimm F, et al. Toward diagnosing *Leishmania infantum* infection in asymptomatic dogs in an area where leishmaniasis is endemic. Clin Vaccine Immunol. 2009;16: 337–343. doi: 10.1128/CVI.00268-08 19129471 PMC2650863

[37] Sangioni LA, Horta MC, Vianna MC, Gennari SM, Soares RM, Galvão MA, et al. Rickettsial infection in animals and Brazilian spotted fever endemicity. Emerg Infect Dis. 2005;11: 265–270. doi: 10.3201/eid1102.040656 15752445 PMC3320454

[38] Latrofa MS, Mendoza-Roldan J, Manoj R, Dantas-Torre F, Otranto D. A duplex real-time PCR assay for the detection and differentiation of *Leishmania infantum* and *Leishmania tarentolae* in vectors and potential reservoir hosts. Entomol Gen. 2021;41: 543–551.

[39] Francino O, Altet L, Sánchez-Robert E, Rodriguez A, Solano-Gallego L, Alberola J, et al. Advantages of real-time PCR assay for diagnosis and monitoring of canine leishmaniosis. Vet Parasitol. 2006;137: 214–221. doi: 10.1016/j.vetpar.2006.01.011 16473467

[40] El Tai NO, El Fari M, Mauricio I, Miles MA, Oskam L, El Safi SH, et al. *Leishmania donovani*: intraspecific polymorphisms of Sudanese isolates revealed by PCR-based analyses and DNA sequencing. Exp Parasitol. 2001;97: 35–44. doi: 10.1006/expr.2001.4592 11207112

[41] Van der Auwera G, Maes I, Doncker S, Ravel C, Cnops L, Van Esbroeck M, et al. Heat-shock protein 70 gene sequencing for *Leishmania* species typing in European tropical infectious disease clinics. Euro Surveill. 2013;18: 20543. doi: 10.2807/1560-7917.es2013.18.30.20543 23929181

[42] Tamura K. Estimation of the number of nucleotide substitutions when there are strong transition-transversion and G+C-content biases. Mol Biol Evol. 1992;9: 678–687. doi: 10.1093/oxfordjournals.molbev.a040752 1630306

[43] Tamura K, Stecher G, Peterson D, Filipski A, Kumar S. MEGA6: Molecular evolutionary genetics analysis version 6.0. Mol Biol Evol. 2013;30: 2725–2729. doi: 10.1093/molbev/mst197 24132122 PMC3840312

[44] Chen H, Li J, Zhang J, Guo X, Liu J, He J, et al. Multi-locus characterization and phylogenetic inference of *Leishmania* spp. in snakes from Northwest China. PLoS One. 2019;14: e0210681. doi: 10.1371/journal.pone.0210681 31022192 PMC6483563

[45] Zhang JR, Guo XG, Chen H, Liu JL, Gong X, Chen DL, et al. Pathogenic *Leishmania* spp. detected in lizards from Northwest China using molecular methods. BMC Vet Res. 2019;15: 1–3. 31818287 10.1186/s12917-019-2174-4 PMC6902407

[46] Mendoza-Roldan JA, Mendoza-Roldan MA, Otranto D. Reptile vector-borne diseases of zoonotic concern. Int J Parasitol Parasites Wildl. 2021;15: 132–142. doi: 10.1016/j.ijppaw.2021.04.007 34026483 PMC8121771

[47] Borja LS, Sousa OMF, Solcà MDS, Bastos LA, Bordoni M, Magalhães JT, et al. Parasite load in the blood and skin of dogs naturally infected by *Leishmania infantum* is correlated with their capacity to infect sand fly vectors. Vet Parasitol. 2016;229: 110–117. doi: 10.1016/j.vetpar.2016.10.004 27809965

[48] Cavalera MA, Zatelli A, Donghia R, Mendoza-Roldan JA, Gernone F, Otranto D, et al. Conjunctival Swab Real Time-PCR in *Leishmania infantum* seropositive dogs: diagnostic and prognostic values. Biology. 2022;11: 1–10. doi: 10.3390/biology11020184 35205050 PMC8869220

[49] Badaró R, Reed SG, Carvalho EM. Immunofluorescent antibody test in American visceral leishmaniasis: sensitivity and specificity of different morphological forms of two *Leishmania* species. Am J Trop Med Hyg. 1983;32: 480–484. doi: 10.4269/ajtmh.1983.32.480 6407345

[50] Paz GF, Rugani JMN, Marcelino AP, Gontijo CMF. Implications of the use of serological and molecular methods to detect infection by *Leishmania* spp. in urban pet dogs. Acta Trop. 2018;182: 198–201. doi: 10.1016/j.actatropica.2018.03.018 29545151

